# Automated classification of midpalatal suture maturation using 2D convolutional neural networks on CBCT scans

**DOI:** 10.3389/fdmed.2025.1583455

**Published:** 2025-06-26

**Authors:** Mahshid Nik Ravesh, Nazila Ameli, Manuel Lagravere Vich, Hollis Lai

**Affiliations:** School of Dentistry, Faculty of Medicine and Dentistry, University of Alberta, Edmonton, AB, Canada

**Keywords:** deep learning, convolutional neural networks, midpalatal suture, CBCT, orthodontics, maxillary transverse deficiency

## Abstract

**Introduction:**

Accurate assessment of midpalatal suture (MPS) maturation is critical in orthodontics, particularly for planning treatment strategies in patients with maxillary transverse deficiency (MTD). Although cone-beam computed tomography (CBCT) provides detailed imaging suitable for MPS classification, manual interpretation is often subjective and time-consuming.

**Methods:**

This study aimed to develop and evaluate a lightweight two-dimensional convolutional neural network (2D CNN) for the automated classification of MPS maturation stages using axial CBCT slices. A retrospective dataset of CBCT images from 111 patients was annotated based on Angelieri's classification system and grouped into three clinically relevant categories: AB (Stages A and B), C, and DE (Stages D and E). A 9-layer CNN architecture was trained and evaluated using standard classification metrics and receiver operating characteristic (ROC) curve analysis.

**Results:**

The model achieved a test accuracy of 96.49%. Class-wise F1-scores were 0.95 for category AB, 1.00 for C, and 0.95 for DE. Area under the ROC curve (AUC) scores were 0.10 for AB, 0.62 for C, and 0.98 for DE. Lower AUC values in the early and transitional stages (AB and C) likely reflect known anatomical overlap and subjectivity in expert labeling.

**Discussion:**

These findings indicate that the proposed 2D CNN demonstrates high accuracy and robustness in classifying MPS maturation stages from CBCT images. Its compact architecture and strong performance suggest it is suitable for real-time clinical decision-making, particularly in identifying cases that may benefit from surgical intervention. Moreover, its lightweight design makes it adaptable for use in resource-limited settings. Future work will explore volumetric models to further enhance diagnostic reliability and confidence.

## 1 Introduction

Maxillary transverse deficiency (MTD) is characterized by a reduced width of the upper jaw relative to the mandible. It can present clinically as posterior crossbite, dental crowding, altered tongue position, and impaired nasal airflow, and has been associated with compromised airway dimensions and increased risk for obstructive sleep apnea (1–8). Treatment options depend heavily on whether the midpalatal suture (MPS)—a key growth site in the maxilla—has fused. In growing patients with an open or partially ossified MPS, non-surgical expansion techniques such as rapid maxillary expansion (RME), slow maxillary expansion (SME), or microimplant-assisted rapid palatal expansion (MARPE) are typically effective (9–13). In contrast, patients with complete MPS fusion often require surgically assisted rapid palatal expansion (SARPE) or segmental LeFort I osteotomy, which carry additional cost, morbidity, and recovery time (14, 15).

Misclassifying the MPS stage can lead to complications such as relapse, pain, and unnecessary surgeries. Therefore, an objective evaluation of MPS maturation is essential for optimizing treatment outcomes and ensuring that patients receive the most effective and least invasive care (16–19). Conventional assessment methods, such as the five-stage CBCT-based classification proposed by Angelieri et al. (Stages A to E), rely on visual interpretation of axial slices and are inherently subjective. Inter-examiner agreement has been reported as low as 43%–68%, highlighting the diagnostic variability that may lead to overtreatment or undertreatment (20–22).

**Figure 1:**
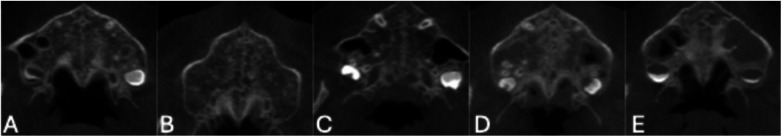
Representative axial CBCT slices showing the five stages of midpalatal suture (MPS) maturation according to angileri et al. **(A)** Stage A: Straight high-density line with no interdigitation. **(B)** Stage B: Scalloped appearance with early interdigitation. **(C)** Stage C: Increased interdigitation and partial fusion. **(D)** Stage D: Significant fusion with reduced suture visibility. **(E)** Stage E: Complete fusion; suture no longer visible.

In Angelieri et al's system as shown in Figure 1, Stage A represents a straight, high-density suture line with no interdigitation (Figure 1A); Stage B shows increased scalloping (Figure 1B); Stage C features two parallel high-density lines (Figure 1C); Stage D marks partial fusion in the palatine bone (Figure 1D); and Stage E indicates complete fusion throughout the palate (Figure 1E), rendering the suture invisible. While this method is widely accepted, staging remains subjective, and clinical decisions often depend not on the specific stage but on whether the suture is *open*, *transitional*, or *fused*. As such, clinicians commonly group these into three actionable categories: AB (immature), C (uncertain), and DE (fused), which directly influence treatment strategy (23).

Recent advances in artificial intelligence (AI), particularly convolutional neural networks (CNNs), have shown promise in automating radiographic interpretation in medicine and dentistry (24–29). CNNs are particularly suited to image classification tasks due to their ability to extract complex features from raw pixel data (30–33). In orthodontics, Zhu et al. (34) recently demonstrated the use of a ResNet-based CNN to classify MPS maturation stages from CBCT images, offering proof of concept. However, ResNet architectures require substantial computational resources and are not easily integrated into routine clinical practice.

Furthermore, prior AI-based studies have generally maintained the five-category structure proposed by Angelieri, which may not align with how clinicians make treatment decisions. In practice, MPS stages are commonly consolidated into three actionable groups:

• **Stages A and B (AB):** immature suture, favorable for non-surgical expansion
• **Stage C:** transitional morphology, uncertain outcome
• **Stages D and E (DE):** fused suture, requiring surgical expansion (23)

Given the importance of early and accurate diagnosis of MTD in ensuring efficient treatment, this study aims to develop and evaluate a 2D CNN model for the automated classification of MPS maturation stages from CBCT images. The model is designed to classify MPS into three groups: AB (stages A and B), C, and DE (stages D and E), corresponding to different treatment strategies. By leveraging a lightweight CNN architecture, this study seeks to balance high classification accuracy with low computational demand, offering a practical tool to improve diagnostic consistency and patient care in orthodontics.

## 2 Materials and methods

### 2.1 Data collection and preparation

This study was approved by the Health Research Ethics Board of the University of Alberta (study number: Pro00125920). Anonymized and de-identified CBCT scans were retrospectively obtained from 155 patients who underwent imaging as part of routine orthodontic treatment between 2014 and 2022 at the University of Alberta Orthodontics Clinic. Patients included in the study were between 7 and 21 years of age. All data were fully anonymized prior to the study, and individual demographic identifiers such as gender were not available. Although CBCT produces volumetric data, all labeling, image selection, and model training in this study were performed exclusively on 2D axial slices.

Scans were excluded if patients had prior orthodontic treatment, impacted upper teeth in the mid-palatal region, congenital craniofacial anomalies (e.g., cleft palate), if the MPS was not clearly visible in a single axial slice, or if image quality was compromised by motion artifacts or scatter. A total of 44 scans were excluded: 34 due to poor visualization of the suture, and 10 due to scatter or artifacts.

The rationale for these exclusions was to ensure that the deep learning model was trained on diagnostically interpretable images, where the MPS could be reliably visualized in a single slice and labeled. Including scans with unclear sutures or confounding anatomy would have introduced label noise, reduced model performance, and undermined study validity. In cases where the presence of the suture was ambiguous, multiple slices were reviewed by orthodontists before exclusion was confirmed. This approach prioritized label accuracy and model generalizability to real-world clinical CBCTs with sufficient image quality for diagnosis.

The remaining 111 CBCT scans from 111 patients were included in the study. Each scan consisted of approximately 450 2D axial slices which were converted from DICOM format to PNG using ITK-SNAP software, resulting in images with a dimension of 726 × 644 pixels. All CBCTs were acquired using a full field-of-view i-CAT scanner (Imaging Sciences International, Hatfield, PA, USA) at medium dimension, with a voxel size of 0.3 mm and an acquisition time of 8–9 s. Axial view of the patient images was classified into 5 groups of MPS maturation stage as first stated by Angeliere et al. by two orthodontists. In case of disagreement, a third orthodontist evaluated the images to determine the class of MPS. Once the patients were classified, the slices showing patients' palates were selected and saved in a separate folder.

The palate slices were then categorized into three groups AB (maturation stages A and B) C and DE (maturation stages D and E). This was done as a way to reduce the amount of variability for the DL model as the aim of this study was to help in reaching a diagnosis for an optimal treatment plan (23).

### 2.2 Data preprocessing

To improve model performance and ensure consistency across samples, several preprocessing steps were applied to the CBCT axial slices prior to CNN training. The goal of preprocessing was to focus the model's attention on the midpalatal suture (MPS), reduce irrelevant variation, and standardize image inputs.

**Figure 2:**
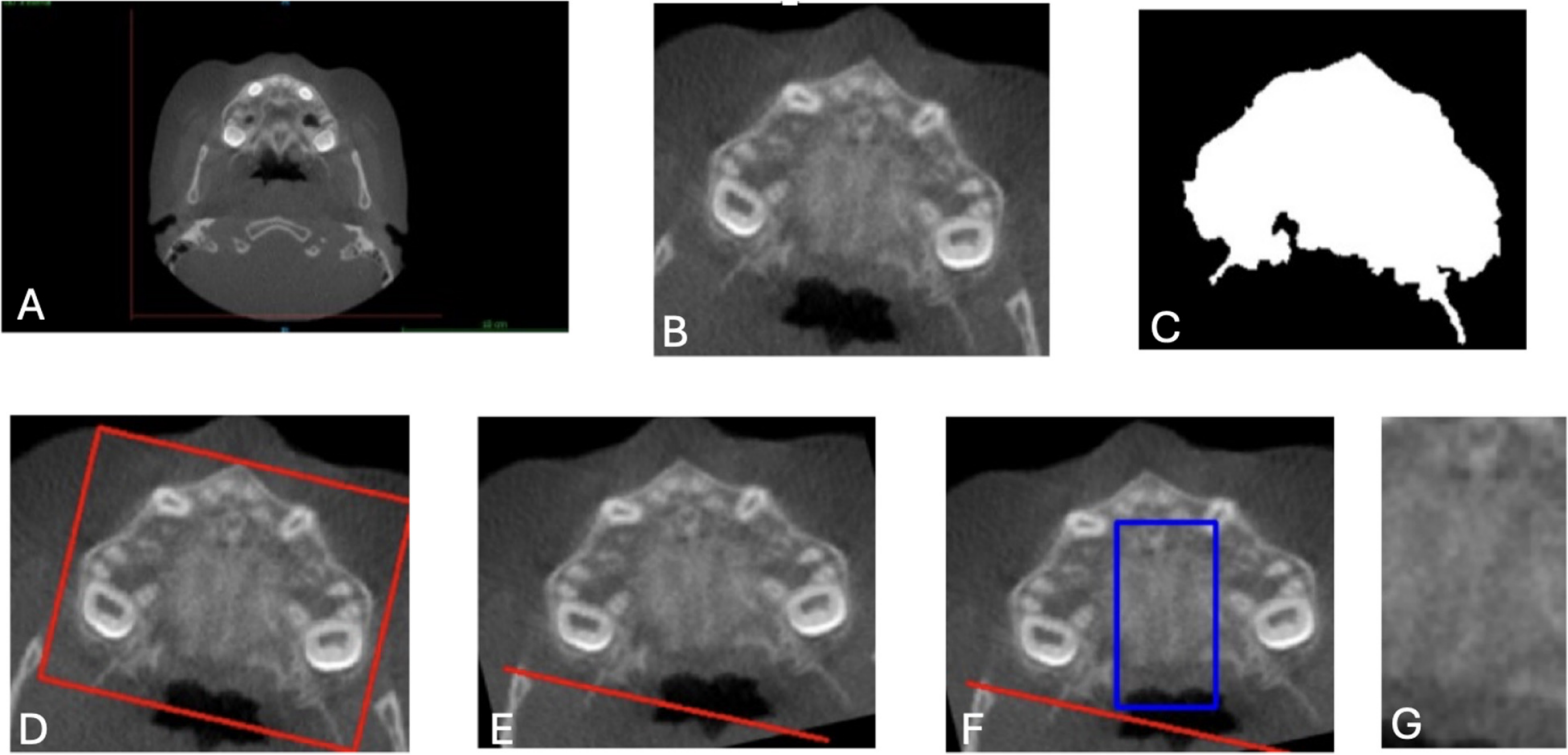
Image preprocessing steps for CNN input preparation. **(A)** Original axial CBCT scan. **(B)** Cropped slice isolating the maxilla using fixed coordinates (190 × 220 pixels). **(C)** Binary mask identifying the maxillary region. **(D)** Angular correction was performed by drawing a rectangle around the widest maxillary contour and estimating the rotation angle. **(E)** Aligned image following rotation to standardize transverse orientation. **(F)** Region of interest (ROI) selection for the midpalatal suture, defined as 140 pixels vertically and 47 pixels horizontally, centered over the MPS. **(G)** Final cropped ROI used as model input. These preprocessing steps were applied uniformly to all axial slices to ensure anatomical consistency, reduce noise, and improve model focus on the midpalatal suture (MPS). CNN, convolutional neural network; ROI, region of interest; CBCT, cone-beam computed tomography.

First, each slice was cropped to isolate the maxilla using fixed predefined coordinates, resulting in a cropped area with dimensions of 190 × 220 pixels (Figure 2B). This ensured that the entirety of the maxilla was included in the Region of Interest (ROI). Next, to further reduce noise and highlight image contours, a Gaussian blur was applied using OpenCV and NumPy libraries (35, 36). A binary mask of the maxilla was also generated for reference (Figure 2C).

To correct angular differences, the images were rotated based on the transverse dimension of the maxilla. A rectangle was drawn around the widest contour (Figure 2D), and the rotation angle was calculated and adjusted to align the images horizontally (Figure 2E). After rotation, the ROI for the MPS was determined by cropping an area 47 pixels around the *X*-axis and 140 pixels around the *Y*-axis, ensuring the MPS was fully captured in all CBCT images. This resulted in a final ROI dimension of 140 × 47 pixels, verified manually for accuracy. (Figures 2F,G).

These cropping dimensions were chosen to capture the MPS consistently across all CBCT images, reducing irrelevant information while maintaining computational efficiency. Each image was manually checked to ensure that the MPS was fully contained within the final region of interest (ROI), thereby maximizing the model's ability to learn relevant features for accurate classification. While this preprocessing pipeline was applied uniformly across all samples, the coordinate parameters and ROI dimensions were based on this specific dataset and may not be directly transferable to other CBCT datasets. Manual verification or dataset-specific adjustments would likely be required when applying the pipeline elsewhere, due to variations in patient anatomy, image acquisition protocols, and field of view. This highlights the importance of validating preprocessing procedures in the context of each new clinical dataset to ensure model reliability and generalizability.

### 2.3 ROI classifier development using DL

To identify the optimal axial slices containing the MPS, a separate binary classification CNN was developed to distinguish between slices that clearly displayed the MPS (preferred) and those that did not (non-preferred).

#### 2.3.1 Data collection

A dataset of 996 axial slices was constructed from the full CBCT volumes of 111 patients. Of these, 575 slices were labeled as “preferred” slices, where the MPS was clearly visible, and 421 non-preferred slices, selected randomly from other regions of the scans.

#### 2.3.2 Data splitting

The dataset was divided into training (70%), validation (20%), and testing (10%) subsets, maintaining class balance (37). This resulted in 402 preferred and 294 non-preferred slices for training, 115 preferred and 84 non-preferred for validation, and 57 preferred and 42 non-preferred for testing.

#### 2.3.3 Model architecture

The architecture consisted of two convolutional layers with 32 and 64 filters respectively, each using a 3 × 3 kernel and ReLU activation. Each convolutional layer was followed by a 2 × 2 max-pooling layer to reduce spatial dimensions and computational load. The output was flattened and passed through a fully connected dense layer with 64 units and ReLU activation, followed by a final dense layer with a sigmoid activation function to output the probability of belonging to the “preferred” class. This compact architecture was designed to balance accuracy with computational efficiency and to minimize the risk of overfitting, given the binary nature of the task and relatively small dataset size.

#### 2.3.4 Training

The model was trained with the Adam optimizer and binary cross-entropy loss function. Early stopping was applied after 5 epochs of no improvement, with the best model weights saved using checkpointing.

### 2.4 2D CNN model for MPS classification

After selecting the preferred slices that include the suture structure using a two-layer CNN model, another 2D CNN was developed to classify MPS maturation stages into three categories: AB (Stages A and B), C, and DE (Stages D and E).

#### 2.4.1 Architecture

The model consisted of nine convolutional layers, designed to progressively extract features from preprocessed axial CBCT slices. The input to the model was a grayscale image of 140 × 47 pixels.

The first convolutional layer applied 64 filters of size 3 × 3, followed by batch normalization, ReLU activation, and max pooling. Each convolutional layer was followed by batch normalization and ReLU activation, and dropout layers (with a rate of 0.5) were interspersed throughout to reduce overfitting.

**Figure 3:**
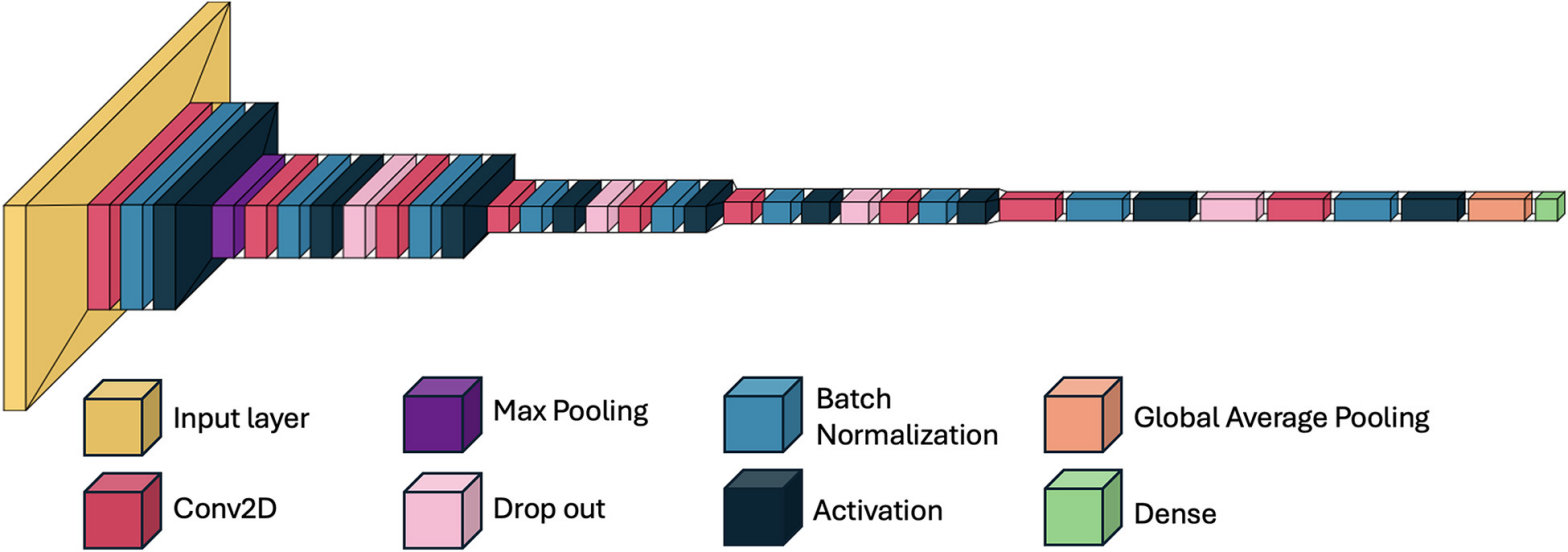
Schematic overview of the 2D convolutional neural network (CNN) architecture for MPS classification. Axial CBCT slices serve as input. Feature extraction is performed through a series of convolutional and pooling layers. Outputs are passed to a fully connected layer and classified into one of three clinically grouped stages: AB, C, or DE. Softmax activation provides class probabilities. MPS, midpalatal suture; CNN, convolutional neural network.

**Figure 4:**
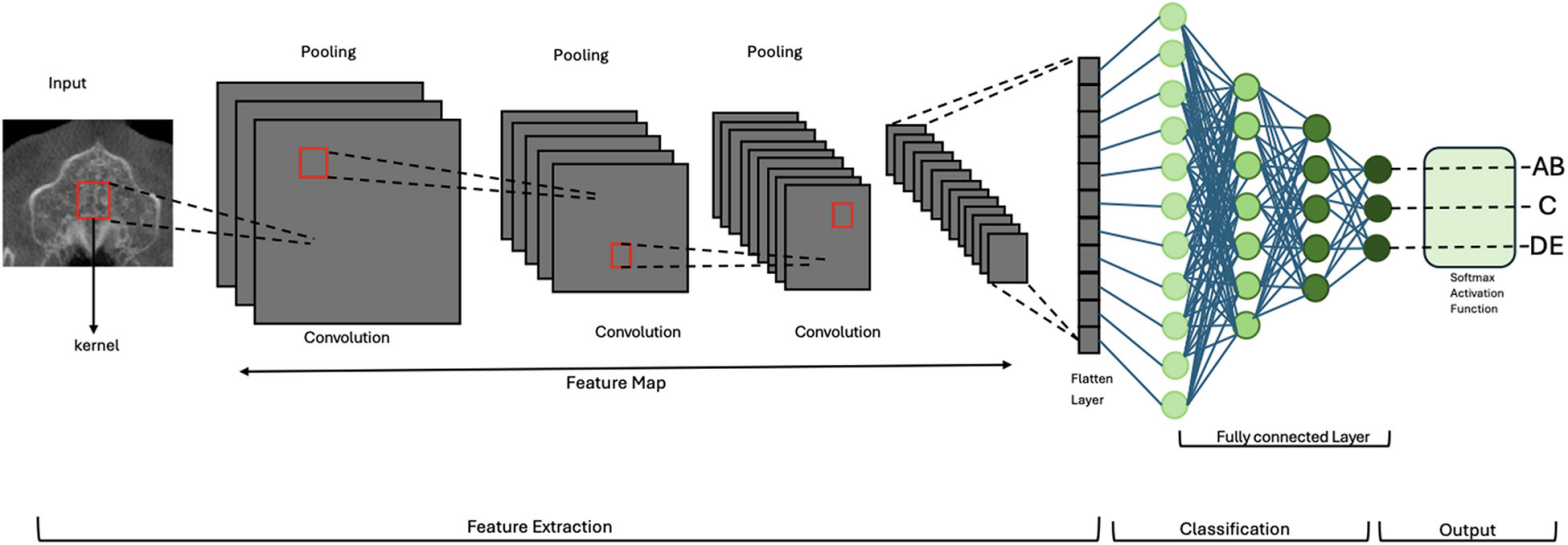
Schematic overview of the 2D CNN used for midpalatal suture maturation classification. The model includes nine convolutional layers, each followed by batch normalization, ReLU activation, and dropout. Max pooling and global average pooling reduce dimensionality before the dense layers. This architecture enabled high classification performance while maintaining low model complexity (4.7 million trainable parameters).

The output from the final convolutional block was passed through a global average pooling layer, followed by a dense layer with three output units and a softmax activation function, which provided the probability distribution across the three classes. The total number of trainable parameters was approximately 4.7 million. The model was implemented using TensorFlow (v2.12) and Keras. A schematic of the architecture is shown in Figures 3, 4.

#### 2.4.2 Training setup

111 CBCT scans from 111 patients were included in this study. The ROI classifier was employed on the CBCTs to create a dataset consisting of 580 images of the ideal slices containing the MPS. The dataset was split for training, validation and test in patient-wise manner, where 70% of the data was used for training, 20% for validation and 10% for testing the CNN architecture.

The model was trained using the Adam optimizer with a learning rate of 0.001, and the categorical cross-entropy loss function. Training was conducted for up to 50 epochs with a batch size of 32. Early stopping with a patience of 5 epochs was applied based on validation loss to prevent overfitting. The best-performing weights were saved for evaluation.

### 2.5 Evaluation metrics

Model performance was evaluated using accuracy, precision, recall, and F1-score metrics for each class (AB, C, DE), providing a comprehensive assessment of the model's ability to classify MPS maturation stages.

To further assess the model's discriminative ability and ranking confidence, one-vs.-rest receiver operating characteristic (ROC) curves were generated for each class. The area under the curve (AUC) was calculated to quantify the model's ability to distinguish each class from the others across a range of probability thresholds.

Additionally, 95% confidence intervals for AUC scores were computed using bootstrapping with 1,000 iterations, providing insight into the statistical reliability of model predictions. All performance metrics were computed on the held-out test set and are reported in detail in the Results section.

## 3 Results

Manual classification of the 111 CBCT by the two orthodontists in this study achieved an inter-rater reliability of 68%, comparably higher than similar studies at 43% (21). Discrepancies were resolved by a third orthodontist with experience in midpalatal suture (MPS) assessment.

### 3.1 ROI classifier performance

A separate CNN model was developed to classify slices as “preferred” (containing the MPS) or “non-preferred.” The ROI classifier was evaluated on a test set comprising 57 preferred images and 42 non-preferred images. The evaluation yielded the following results, with a test accuracy of 99%. During the training and validation phases, both training and validation accuracies reached 100%, accompanied by negligible loss values, indicating effective model training. Manual verification of the model-selected slices confirmed agreement with expert selection in 99% of cases. This enabled construction of a final dataset of 580 ideal axial slices, used for MPS stage classification.

### 3.2 CNN model training and validation

The CNN model was trained to classify midpalatal maturation stages based on CBCT images. The input shape was set to 140 × 47, with a total of 405 training samples categorized into three groups: AB (127 samples), C (158 samples), and DE (120 samples).

The training utilized early stopping with a patience of 15 epochs and model checkpointing to save the best-performing weights. Initially set for 100 epochs with a batch size of 16, the training concluded at 90 epochs due to no improvement in validation loss after epoch 75. At that point, the model achieved a training accuracy of 97.81%, a validation loss of 0.0626, and a validation accuracy of 98.08%. The training duration for this epoch was approximately 5 s, with a processing time of 218 ms per step.

### 3.3 Testing results

**Figure 5:**
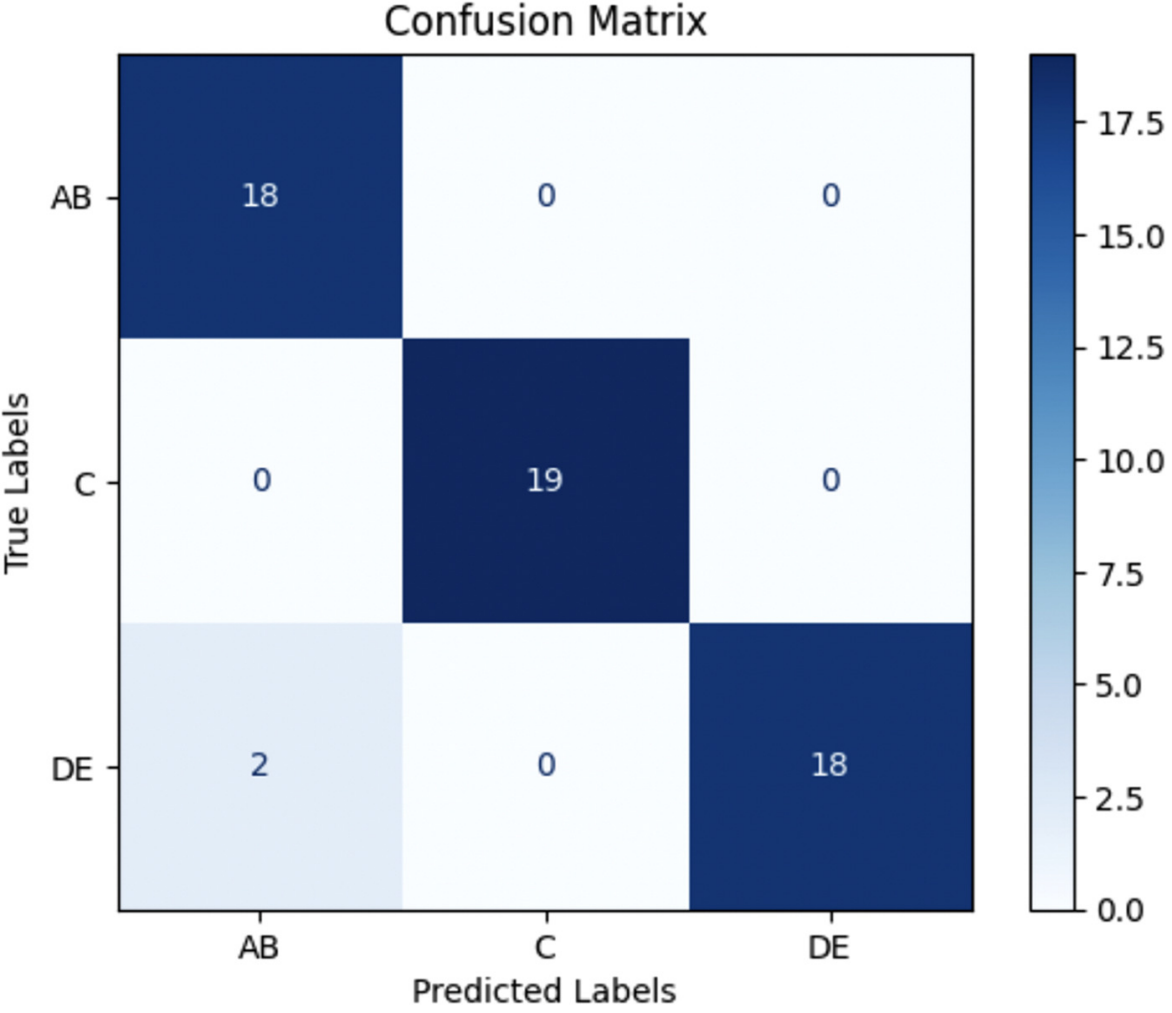
Confusion matrix for 2D CNN model testing results. The model achieved a test accuracy of 96.49%. It classified AB with 100% recall (18/18), C with 100% recall (19/19), and DE with 90% recall (18/20). Misclassifications primarily occurred between DE and AB. AB = Stages A and B; DE = Stages D and E.

The trained model was evaluated on a separate test dataset consisting of 57 images, distributed among the three groups: AB (18 samples), C (19 samples), and DE (20 samples). The testing results revealed a test loss of 0.2286 and test accuracy of 96.49%. The classification performance was further assessed using precision, recall, and F1-score metrics for each class (AB, C, DE) as demonstrated in Table 1 and Figure 5.

**Table 1:** Classification performance metrics (precision, recall, and F1-score) for each class (AB, C, DE).

Class	Precision	Recall	F1-Score
AB (18)	0.90	1.00	0.95
C (19)	1.00	1.00	1.00
DE (20)	1.00	0.90	0.95

### 3.4 ROC and AUC analysis

**Figure 6:**
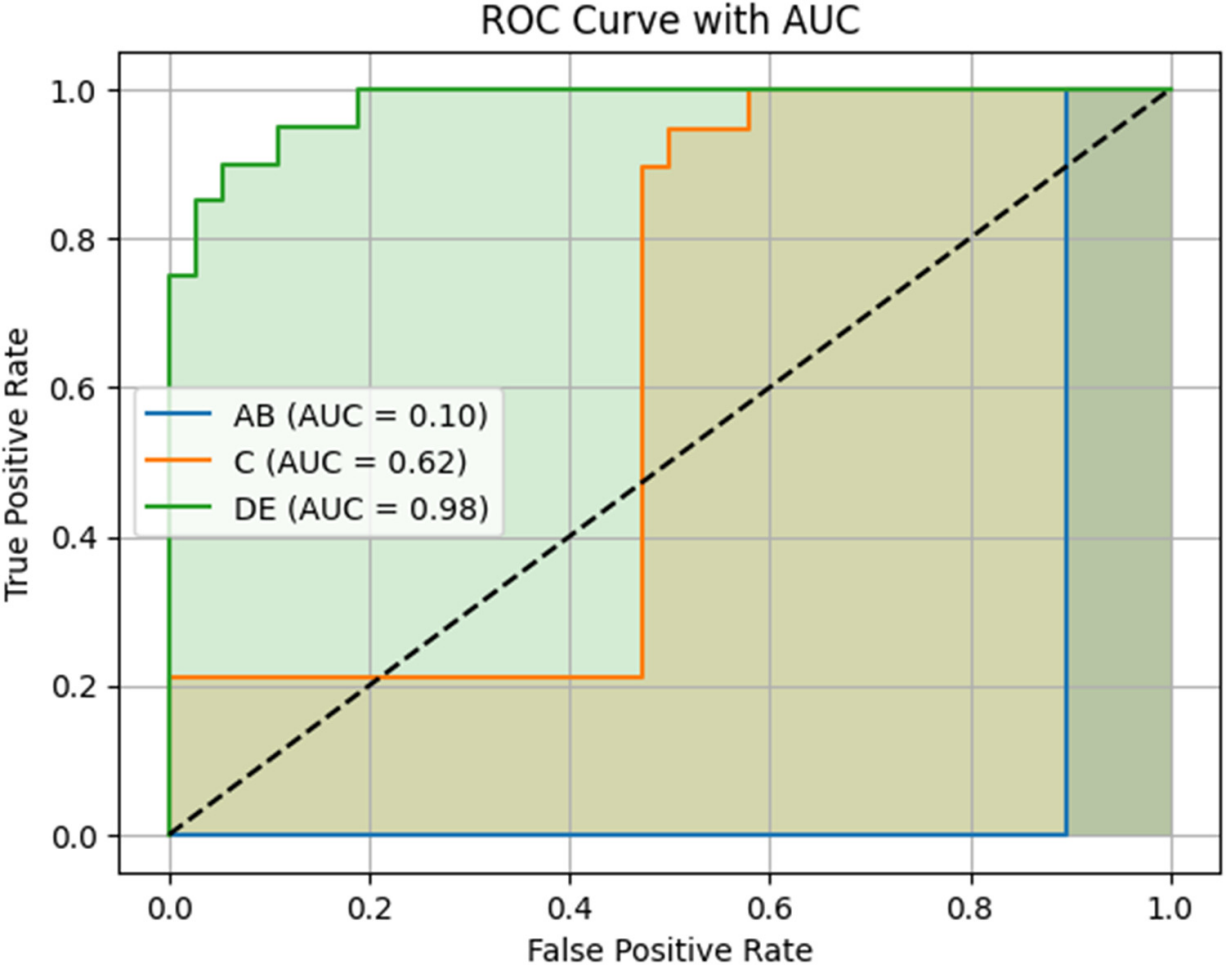
Receiver operating characteristic (ROC) curves and area under the curve (AUC) scores for the three MPS maturation classes. The model's performance is shown using a one-vs.-rest approach. The DE class achieved a high AUC of 0.98, indicating excellent discriminative ability and model confidence. In contrast, the AB and C classes had lower AUC values (0.10 and 0.62, respectively), reflecting reduced certainty in predictions, particularly in early-stage classification. This may be due to anatomical overlap and subtle feature transitions between early and intermediate maturation stages. AB = Stages A and B; DE = Stages D and E.

ROC curves were generated for each class using a one-vs.-rest approach. The model achieved an AUC of 0.98 for class DE, indicating perfect separation from the other classes. The AUCs for AB and C were 0.10 and 0.62, respectively. Full ROC curves and associated metrics are shown in Figure 6.

## 4 Discussion

In this study, a dataset of 580 CBCT slices containing the MPS was used to train a lightweight 2D CNN for automated MPS maturation stage classification into three clinically relevant groups: AB, C, and DE. The proposed CNN achieved a training accuracy of 97.81%, a validation accuracy of 98.08%, and a test accuracy of 96.49%. Class-wise performance metrics demonstrated high precision (AB: 0.90, C: 1.00, DE: 1.00) and recall (AB: 1.00, C: 1.00, DE: 0.90), with F1-scores ranging from 0.95–1.00. These results suggest that our CNN architecture effectively supports early detection of MPS fusion stages from axial CBCT images. Clinically, the AB, C, and DE stages also broadly correspond to pre-pubertal, pubertal, and post-pubertal phases of growth, respectively, which are key considerations in determining the timing and method of maxillary expansion.

ROC analysis revealed AUC values of 0.10 for AB, 0.62 for C, and 0.98 for DE. While DE classification was highly confident and well-separated, the comparatively lower AUCs for AB and C suggest that the model had more difficulty distinguishing these early and transitional stages with consistent confidence. This may reflect the anatomical overlap and subtlety between AB and C stages, which are often difficult to distinguish even among experienced clinicians. In our dataset, inter-rater agreement between two orthodontists was 68%, underscoring the inherent subjectivity and variability in labeling these stages. Although the model predicted AB and C correctly in most cases—as reflected in the high F1-scores—the ROC-derived AUC indicates that the model often assigned similar probabilities across neighboring classes, reducing its overall ranking confidence.

Our results can be compared to Zhu et al., who also employed a CNN-based approach (ResNet18) for MPS classification from CBCT images where the distinction in model complexity is worth noting. Zhu et al.'s ResNet18 model comprises 11.69 million parameters, trained from 785 patient samples, whereas our custom model contains only 4.7 million parameters—approximately 60% fewer parameters. Despite this reduction in complexity, our model achieved significantly higher training accuracy (97.81% vs. 79.10%), underscoring the efficacy of a more efficient, lightweight architecture in this classification task. Our model demonstrates alternative approaches in development of small and task-specific DL models that can be applied in resource-constrained environments, such as clinical settings with limited samples, computational power, or on mobile devices.

Our findings align with previous research highlighting the effectiveness of DL techniques, particularly CNNs, in medical image analysis. Other studies have demonstrated CNNs' ability to interpret complex biomedical images effectively, including tasks like interstitial lung disease pattern classification and dental diagnostics. These studies further validate the utility of CNNs in healthcare, particularly in automating diagnostic processes and reducing human error (25, 26, 38–40).

Moreover, the lightweight nature of our CNN architecture offers practical advantages, especially in orthodontic clinics where computational resources may be limited. The model's efficiency, coupled with its high accuracy, demonstrates its potential for real-world applications, where rapid and accurate assessment of midpalatal suture maturation could significantly enhance treatment planning.

However, it is important to acknowledge some limitations of our study. While the sample size of CBCT images aligns with typical practices in medical research, it may impact the generalizability of our findings to larger and more diverse populations.

Future studies should focus on evaluating the model's performance with larger and more diverse datasets to ensure its robustness and applicability across different demographics.

A limitation of the approach used in this study was the exclusion of midpalatal sutures that were not visible in a single slice, as the methods used in the study can effectively classify, albeit only with individual slices. Future research should explore three-dimensional (3D) approaches to provide a more comprehensive analysis of MPS maturation stages, as well as optimize imaging protocols or employ AI-based enhancement techniques to improve suture detectability and reduce the need for image exclusion.

Another limitation of this study is the higher radiation dose associated with CBCT compared to occlusal radiographs. However, CBCT offers significantly greater image quality and precision, which was essential for the development of our small models (41). The ability to use smaller models stems from the superior clarity provided by CBCT, allowing for more precise visualization and classification. While occlusal radiographs were previously used to visualize the MPS (42), their clarity and accuracy were limited, as they provided a less detailed view of a potentially thin structure embedded deep within the palatal bone, making classification more challenging (43). As small model development becomes a growing focus in the field, the enhanced resolution of CBCT imaging will continue to play a crucial role in advancing research and clinical applications.

It is critical to recognize that our findings rely on Angelieri's staging system for midpalatal suture maturation, which could affect the validity of our results if future studies challenge this classification's accuracy. Additionally, building on this work, future research will explore volumetric (3D) CNN models to capture full spatial context. Preliminary results from our follow-up study suggest improved AUC values for AB and C stages, supporting the potential of 3D architectures to enhance diagnostic confidence in. By incorporating advanced imaging techniques and larger datasets, we aim to further enhance the accuracy and clinical relevance of AI-driven tools in orthodontic diagnosis and treatment planning.

## 5 Conclusion

In conclusion, this study developed a lightweight 2D convolutional neural network (CNN) to automate the classification of midpalatal suture (MPS) maturation stages from axial CBCT slices. The proposed model demonstrated high accuracy, with strong precision and F1-scores across all three clinically grouped classes: AB, C, and DE. ROC analysis further confirmed excellent performance for the DE stage, which is critical for identifying cases that may require surgically assisted expansion.

The model's efficient architecture, combined with its robust classification ability, highlights its potential for real-world clinical use—particularly in orthodontic settings where rapid and reliable MPS assessment could aid treatment planning. While classification of early and transitional stages (AB and C) presented lower AUC values, these findings reflect known diagnostic ambiguity and suggest areas for further development. Future research will explore 3D CNN models to improve diagnostic confidence and generalizability through volumetric context and larger, more diverse datasets.

## Data Availability

The data analyzed in this study is subject to the following licenses/restrictions: The dataset consists of patient CBCT images which are the property of Dr. Manuel Lagravere Vich from University of Alberta, School of Dentistry, Orthodontics Clinic. Requests to access these datasets should be directed to manuel@ualberta.ca.
